# A critical realist evaluation of an integrated care project for vulnerable families in Sydney, Australia

**DOI:** 10.1186/s12913-020-05818-x

**Published:** 2020-10-31

**Authors:** E. Tennant, E. Miller, K. Costantino, D. De Souza, H. Coupland, P. Fotheringham, J. Eastwood

**Affiliations:** 1 Healthy Homes and Neighbourhoods Integrated Care Initiative, Community Health Services, Sydney Local Health District, 24 Liverpool Road, Croydon, NSW 2132 Australia; 2 School of Public Health and Community Medicine, The University of New South Wales, Kensington, NSW 2052 Australia; 3 Centre for Research in Education, Torrens University Australia, Flinders Street Campus, Melbourne, VIC 3000 Australia; 4 Drug Health Services, Royal Prince Alfred Hospital, Level 6, King George V Building Missenden Road, Camperdown, NSW 2050 Australia; 5 Discipline of Addiction Medicine, Faculty of Medicine and Health, The University of Sydney, Camperdown, NSW 2050 Australia; 6 Population child health group, School of Women’s and Children’s Health, The University of New South Wales, Kensington, NSW 2052 Australia; 7 Ingham Institute for Applied Medical Research, 1 Campbell St, Liverpool, NSW 2170 Australia; 8 Charles Perkins Centre, Menzies Centre for Health Policy, Discipline of Child and Adolescent Health, Central Clinical School, and School of Public Health, University of Sydney, Sydney, New South Wales 2006 Australia; 9 Sydney Institute for Women Children and their Families, Camperdown, NSW 2050 Australia; 10 Community Paediatrics, Croydon Community Health Centre, 24 Liverpool Road, Croydon, NSW 2132 Australia

**Keywords:** Integrated care, Critical realism, Design, Engagement

## Abstract

**Background:**

Healthy Homes and Neighbourhoods (HHAN) Integrated Care Initiative was established to improve the care of families with complex health and social needs who reside in Sydney Local Health District. HHAN seeks to provide long-term multi-disciplinary care coordination as well as enhance capacity building and promote integrated care. The critical realist study reported here is part of the longitudinal development and evaluation of complex integrated health and social care interventions in Sydney, Australia.

**Methods:**

We describe the qualitative component of a critical realist pilot case study aimed at exploring, explaining and refining emerging HHAN programme theories in relation to care coordination. Qualitative interviews were undertaken with HHAN clients (*n* = 12), staff and other stakeholders (*n* = 21). Interviews and coding used a context (C), mechanism (M) and outcome (O) framework. Inductive, deductive, retroductive and abductive modes of reasoning were used with the CMO heuristic tool to inform the developing programme theory.

**Results:**

The mechanisms underpinning effective engagement of clients by care coordinators included: building trust, leveraging other family, social and organisational relationships, meeting clients on their own terms, demonstrating staff effectiveness as quickly as possible, and client empowerment. Mechanisms for enhancing care integration included knowledge transfer activities and shared learning among collaborators, structural and cultural changes, enhancing mutual respect, co-location of multidisciplinary and/or interagency staff and cultivating faith in positive change among staff.

**Conclusions:**

Use of a critical realism case study approach served to elucidate the varied influences of contexts and mechanisms on programme outcomes, to highlight what works for whom and in what context. Findings supported the initial programme theory that engagement and trust building with clients, alongside enhanced collaboration and integration of services, improved outcomes for vulnerable families with complex needs. Further research is needed to explore the cost-effectiveness of integrated care initiatives, in view of the long term nature of service provision and the risk of staff burnout.

**Supplementary information:**

The online version contains supplementary material available at 10.1186/s12913-020-05818-x.

## Background

Social disadvantage is a multi-dimensional concept, reflecting a range of indicators tied to financial resources, social capital, social exclusion, health and education outcomes [1, 2]. Disadvantaged families often suffer from the convergence of adverse social, mental and physical health issues that can be overwhelming. Data shows that social disadvantage tends to concentrate geographically [3] and can create intergenerational cycles of trauma and disadvantage [4].

Unfortunately, disadvantaged families can easily become invisible to health and social service providers and policy makers. Social welfare programs designed to provide health and social assistance to those in need are subject to an “inverse care law” whereby the most disadvantaged are most likely to miss out on the care they need [5]. Australian children and families who are disadvantaged are subject to increased rates of inequities in health and wellbeing [6]. This, in part, relates to access to care. Data from the Organisation for Economic Cooperation and Development (OECD) countries shows that the least advantaged members of society are least likely to see a specialist [7] and this is more pronounced in countries that have some privatisation of the healthcare system [7]. Socio-economically disadvantaged Australian women are less likely to access specialist, dental and allied health services compared with other strata in society [8].

Often a multi-agency approach is required to improve health outcomes, given the complex nature of problems [9]. There has been increasing recognition that services need to be reoriented to be “fit for purpose” in assisting vulnerable clients by addressing the social determinants of health [10]. In addition, access to services should promote equity and be cost effective in preventing the consequences of poor physical and mental health, and adverse social outcomes. An emerging evidence base, both internationally and in Australia, supports the adoption of integrated care initiatives to make services, support and care more accessible for vulnerable families with complex needs [11–13].

Goodwin observes that there are many definitions of integrated care and argues that the principles of integrated care should extend to the wider definition of promoting health and well-being [14]. Singer and colleagues define integrated care into two dimensions, namely, patient-centeredness and care coordination [15]. Their definition emphasises patients’ central role as “active participants in managing their own health by including patient centeredness as a key element of integrated patient care”. Of relevance to the study reported here, an ecological model of integrated care has been advanced by Wollcott and colleagues that highlights the importance of considering the person-centred care in terms of social networks and social ecology [16]. The WHO integrated people-centred health services (IPCHS) framework advances the vision in which “all people have equal access to quality health services that are co-produced in a way that meets their life course needs, are coordinated across the continuum of care, and are comprehensive, safe, effective, timely, efficient and acceptable; and all carers are motivated, skilled and operate in a supportive environment [17]. In the context of study reported here, the term “integrated care” is used for a person-centred approach to the delivery of effective, efficient and well-connected care that is organised around a person’s health and social care needs.

In response to concern regarding the needs of vulnerable families, the Inner West Sydney health, education and social agencies collaborated in 2013 to design a range of interagency initiatives [18]. That collaborative design was informed by a translational social epidemiology perspective and related theory building [19–30]. The translational social epidemiology approach is inspired by the challenge by Muntaner [31] for social epidemiologists to move from the study of causal mechanisms toward the generation of social interventions in partnership with affected populations [32, 33]. In 2014, collaborative interagency work commenced on an Inner-West Interagency Child Health and Well-being Plan [34]. Following the launch in 2014 of a New South Wales (NSW) Government integrated care initiative, the Healthy Homes and Neighbourhoods (HHAN) Integrated Care Initiative was designed [35]. The HHAN initiative aims to break intergenerational cycles of disadvantage and trauma within affected communities in Sydney Local Health District (SLHD). The HHAN Theory of Change is at Additional file 1, the Initial Programme Theory is at Additional file 2 and the initial Logic Model is at Additional file 4.

The Healthy Homes and Neighbourhoods Integrated Care Initiative uses a stratified population-based approach to address the needs of families who are experiencing adversity, while supporting parallel interventions for families more generally. The approach to identifying the most vulnerable families who are disconnected from key services has been developed using existing perinatal risk assessment systems, and involves developing new cross-agency assessment and referral pathways, and improved hospital recognition of the needs of families using an e-health solution.

“The initiative has the following key features:

1. Multiple core and non-core agencies working together over a sustained period of time (i.e. 5 years) with families with complex health and social needs
2. Co-design and co-production of the initiative in partnership with families and service partners
3. All the needs of enrolled families are in scope for the intervention, including housing, employment, income support and legal advice
4. An early intervention and public health approach to interrupting cycles of family disadvantage, poor health and psychological trauma
5. A focus on efficiency through the maximum use of, and leverage from, existing family, societal and government resources, including Medicare scheduled services
6. Use of evidence-informed integrated care methods by service partners, including family case conferencing, and ‘wrap-around’ care delivery
7. Encouraging families to have a ‘health home’ for all their health needs and supporting progress towards self-efficacy
8. Providing a supporting structure to general practice providers to care for families that are often seen to be ‘too difficult’
9. Development and implementation of shared assessment tools and referral criteria
10. Implementation of family assessment and engagement tools that can be used over the long-term to monitor the health and wellbeing of family members

A central element of the initiative is targeted long-term sustained cross agency care coordination. The design acknowledges the need for significant system redesign and commitment from partners. The initial model required a care coordination team with both project-funded and partner-funded components as a means of ensuring sustainable ‘collaboration’. The initiative also includes local elements through deliberate recruitment of families and service partnerships in the City of Canterbury and City of Sydney local government areas. This last component enabled the development of ‘demonstration-site’ place-based partnerships with local general practice, schools, family support agencies, local government, religious and faith-based organisations and community members.” [36].

This manuscript reports the qualitative findings of a pilot evaluation of HHAN, focusing on the care coordination component of the programme, using a critical realist case study approach. Realist evaluation draws on principles of critical realism and social theory [36–39]. The approach can be readily applied to the discipline of translational social epidemiology for the purpose of evaluating complex interventions such as HHAN [25] and developing implementation and programme theories [40].

Critical realism acknowledges that as well as observable phenomena, there are also unobservable forces at play that produce events under certain conditions and create the mechanisms (M) and conditions or contexts (C) that produce outcomes (O). Rather than approaching programme evaluation by simply examining outcomes for those who participated and those who did not, critical realist evaluation examines Context-Mechanism-Outcome (CMO) configurations to assess what works, for whom and in what context [38]. Furthermore, success often depends on contextual factors and assessment of these is crucial in determining whether translation of an initiative to other settings will work [41].

The manner in which this change manifests within a context results in programme outcomes. The identification of generative mechanisms and counteracting mechanisms requires consideration of context. De Souza [42] conceptualised context, in a social program, as comprising aspects of structure, culture, agency and relations (Table 1).

**Table 1:** Context, Mechanisms, Outcomes, modified from [42]

Context	Mechanisms related to	Outcomes
Structure - Institutional/Organisational	roles, practices, resources, processes	(T), (I) or (R) of institutional/organisational structure
Culture - Institutional/Organisational	group ideas and propositional formulations about the institution/organisation	(T), (I) or (R) of institutional/organisational culture
Agency	individual beliefs and reasons for actions or non-action	(T), (I) or (R) of individual agency within the institution/organisation
Relations	maintaining, adjusting or redistributing power/duties/responsibilities	(T), (I) or (R) of institutional/organisational relations

Note. Transformation (T): indicates mechanisms related to different parts of context, and activated by the social program, are producing some anticipated/desired outcomes

Invariance (I): indicates that mechanisms related to different parts of context, undergo changes in attributes irrelevant to transformation or reproduction of the context (e.g. doctor-patient relation remains, though different persons may fill the roles)(cf. Sayer, 1992)

Reproduction (R): indicates relevant mechanisms related to different parts of context have not been adequately activated by the social program, thereby reproducing outcomes the social program aimed to change

What constitutes a mechanism has received much debate and critique, leading to various constructs of a mechanism when applied in realist review and evaluation practice. Critical realists tend to use the term ‘mechanism’ in relation to effects, and ‘powers’ in relation to structures. Collier [43, p 60] highlights this relationship by stating that “Effects are ascribed to causal powers, causal powers to inner structure (and place in larger structures) of the causal agent”. Greenhalgh et al. describe mechanisms as “underlying changes in the reasoning and behaviour of participants that are triggered in particular contexts” [41]. Westhorp [44] has argued that different constructs of mechanisms are required if they are to align with the principles of Critical Realism, and identified five constructs of mechanisms, namely: 1) powers and liabilities; 2) forces; 3) interactions; 4) feedback and feedforward processes; and 5) reasoning and resources. This study adopts a critical realist understanding of mechanisms which describes them as “that which can cause something in the world to happen, and in this respect mechanisms can be of many different kinds” [45].

**Fig. 1:**
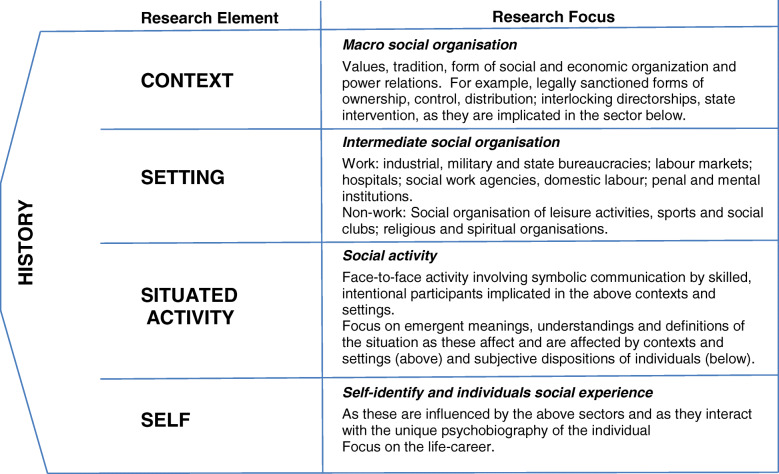
Realist Research Map [47]

Critical realism considers reality to be layered with level-specific mechanisms within what Bhaskar and Danermark called a laminated system [46]. For this purpose the layered levels of: Self, Situated Activity, Intermediate Level and Macro Level, as proposed by Layder [47] were used (Fig. 1).

The multi-facetted complexity of the HHAN integrated care initiative required a more nuanced approach than is usually used for a simple programme evaluation. The HHAN critical realist initial programme theory [35] was developed using a Context (C), Intervention (I), Mechanism (M), Outcome (O) (CIMO) configuration as proposed by Denyer and colleagues [48]. The resulting HHAN initial programme theory uses both the CIMO heuristic and ontological layering proposed by Layder (Additional file 2). In this manuscript we will use, however, the more limited CMO heuristic.

## Methods

**Fig. 2:**
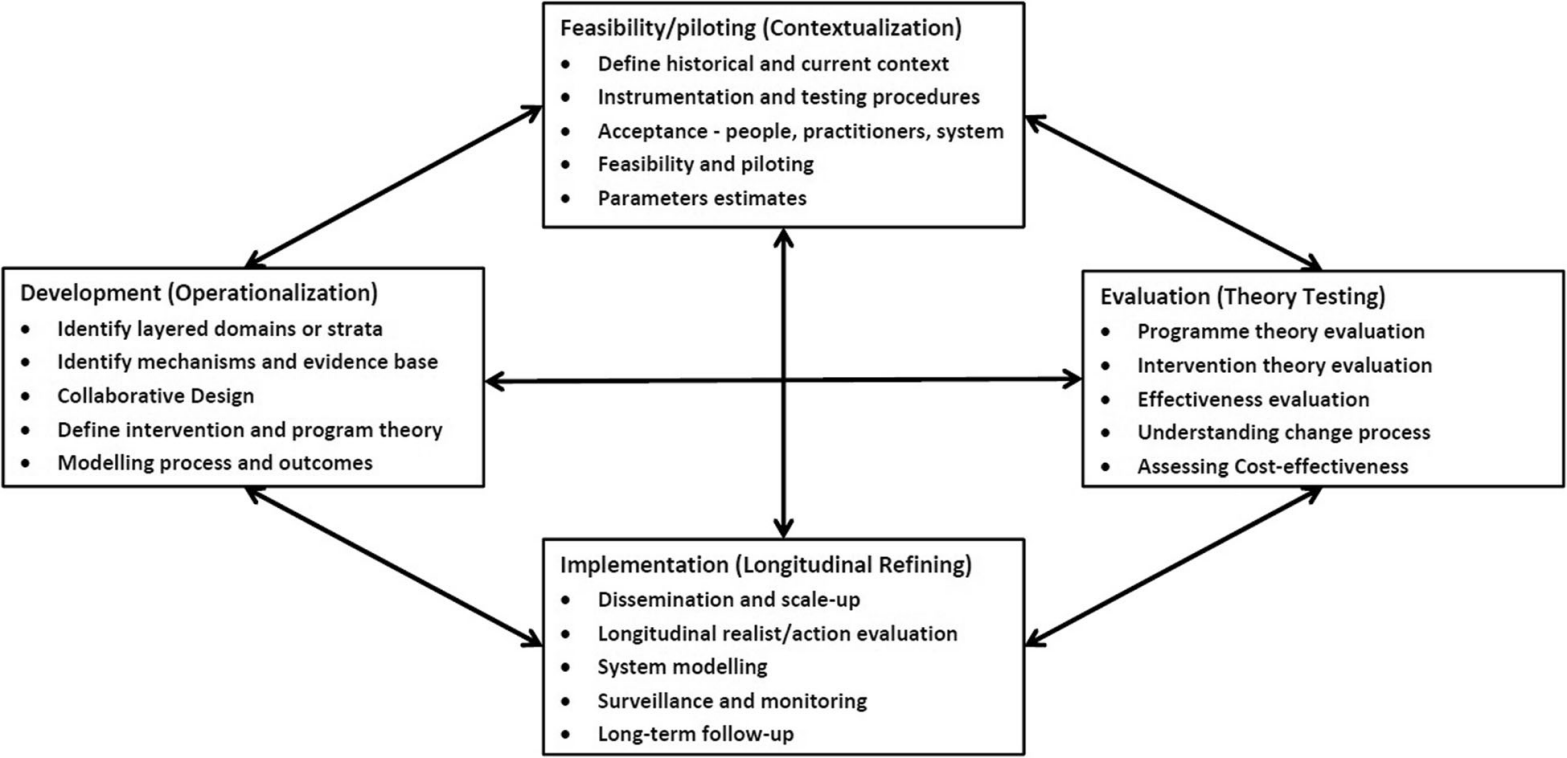
Key elements of the HHAN development and evaluation process [36], adapted from Craig et al. 2008 [49]

As a pilot realist case study, the research aimed to explore emerging HHAN programme theories and propositions in relation to care coordination. Through qualitative data collection, CMO configurations for key HHAN programme components were developed in order to explain and refine the initial programme theories and for future service evaluations. The study forms part of a larger programme of continuous research, design and evaluation [36], informed by the UK Medical Research Council (MRC) Framework for evaluating complex health interventions of 1) development, 2) feasibility/piloting, 3) evaluation and 4) implementation. The framework has been adapted to include: critical realist, theory driven, and continuous improvement approaches (Fig. 2) [36].

A qualitative approach was used to explore how the theorised HHAN programme mechanisms manifested in concrete situations. Through preliminary discussions with the program’s architects, involved stakeholders and enrolled clients and on reviewing the program theory (Additional file 2), an interview guide was developed based around exploring the contexts, mechanisms and outcomes relating to the program (Additional file 3). Through the preliminary analysis, it became evident that clients were engaged with HHAN for very diverse reasons, all falling under the umbrella of vulnerability. Similarly, stakeholder organisations have varying roles and mandates when assigned to clients. For this reason, it became clear that broad sampling to encompass multiple perspectives was required in order to fully understand and evaluate the service. Thirty-three semi-structured interviews were conducted with HHAN clients or members of their family networks (*n* = 12), and service providers (*n* = 21).

Purposive sampling in a number of HHAN programme sites was used to recruit a sample that reflected a range of perspectives [38]. The HHAN program is community-based and district-wide. The Theory of Change (Additional file 1) focuses on service packages and local services that are individually tailored to each individual client’s needs. The program therefore encompasses clients situated in varying geographical locations within the health district. The challenges experienced by families in one geographical area have been found to differ significantly from those experienced in others, despite these families co-existing within one city. The reasons for this include factors such as cultural factors, provision of local services, setup and organisational culture within those services, and density of housing. Stakeholder organisations include primary, secondary and tertiary care agencies as well as governmental and non-governmental organisations. Staff from these organisations offer varying perspectives coloured by personal experience and organisational culture. When sampling for interviewees, the study team attempted to include clients and stakeholders from as many of these diverse groups as possible. During the development of the project, the study team became aware that there were some stakeholders with opposing views. These stakeholders were deliberately interviewed to obtain a variety of perspectives about the program. The interviewer was transparent in informing interviewees that those with opposing views were also being approached, a strategy which has been reported to encourage interviewees to consider the program more deeply than from their own perspective [50].

Potential participants were initially contacted by the HHAN staff working with them. If the client or family member consented to be interviewed, the investigators followed up face to face, by phone or email. All client participants gave written consent to be involved in the study and received a $50 supermarket voucher to thank them for their time. Ethics approval for the study was granted by the Research Ethics and Governance Office, The Royal Prince Alfred Hospital, Sydney Local Health District (X15–0138 & HREC/15/RPAH/190).

An interview guide (Additional file 2), designed to explore contexts, mechanisms and outcomes relating to the program [51] was developed to guide the interviews. Interviews were conducted by the lead author (ET) at the participant’s home, a care facility or place of work. While the researcher was unknown to clients, she had a working relationship with HHAN staff who participated in interviews. Questions explored reasons why participants got involved with HHAN, how the service helped, reasons why HHAN did or did not work well, how clients’ relationships with their children changed, and how and why their utilisation of other services had changed. Interviews lasted between 30 and 90 min and were audio-taped and then professionally transcribed.

Interview transcripts were de-identified and imported into NVivo version 11 for thematic analysis. Line by line coding of each transcript was used to define the underlying meanings. A coding template was developed based on analysis of five interviews. The process was iterative, with further codes being added as necessary with ongoing data review and theory refining. Three reviewers worked independently and then met to discuss findings and merge and collapse categories.

Programme propositions were expressed in realist terms as Context, Mechanism and Outcome (CMO) configurations, including the contextual levels of Self, Situated Activity, Intermediate Level and Macro Level, as proposed by Layder [47] (Fig. 1). CMO configurations were developed through iteration and by applying inductive, retroductive and abductive modes of reasoning [52]. Theories were then organised connecting the outcomes with identified mechanisms that were triggered to generate those outcomes, and finally the contexts within which those mechanisms were triggered. Once outcomes were identified in the first instance, mechanisms and contexts linked to outcomes were generated. The pilot phase analysis described here, used the first three steps of a five step abductive reasoning approach. The five step abductive reasoning approach for theory building and evaluation, draws on the work of Bygstad and Munkvold [53] and includes: 1) the initial programme theory; 2) identification of candidate mechanisms for different outcome scenarios; 3) retroductive thinking to link mechanisms to relevant context; 4) judgemental rationality to evaluate and compare explanations; and 5) theoretical abstraction – retrodiction applied to multi-case explanations.

## Results

### Sample characteristics

All client participants (*n* = 12) were female caregivers. A quarter were grandmothers who had assumed responsibility for a child under kinship care and the remainder were birth parents. Almost half the client participants (5/12) identified as single parents. Almost all client participants experienced mental health issues (11/12) and significant relationship difficulties (11/12).

Service provider participants (*n* = 21) came from a range of backgrounds, including medicine/nursing (*n* = 7), education (n = 2), specialist case workers (*n* = 6) and social work (n = 6). Four service providers were HHAN staff. However, the majority (*n* = 17) were stakeholders from HHAN partner organisations, including General Practice (*n* = 4), New South Wales (NSW) Family and Community Services (FACS) (n = 4), the NSW Department of Education (*n* = 2) and non-government organisations (NGO) and charities (*n* = 5).

### CMO configurations

Analysis of the Interview data identified two distinct groups of CMO configurations. These two groups of CMOs related to two ontological strata, as defined by Layder, and incorporated in the HHAN initial programme theory, namely: 1) situated or face-to-face activity - engagement of vulnerable clients; and 2) intermediate level social and service organisation – integrating care. The CMO configurations will be presented here using these two ontological layers.

#### Strata: situated or face-to-face activity - engagement of vulnerable clients

In order to improve the care of vulnerable clients, one must work in partnership with them. The care coordination aspect of HHAN revolved around the ability of the care-coordinators to engage this group and gain their trust. Five CMO configurations related to engagement of vulnerable clients were identified.

##### Leveraging other relationships (Table 2)

The concept of “wrap-around care” was a key design element of the programme theory [36] operating predominantly at the contextual Situated Activity (face-to-face activity) level. HHAN care-coordinators often tried to engage with the spouse, children and grandparents to provide support to the whole family.

**Table 2:** CMO configuration “Leveraging other relationships”

Contextual factors	Mechanism	Outcomes
Presence of domestic violence	Leveraging of pre-existing relationships to provide wrap-around care and engage clients with additional services	Clients’ improved access to care and engagement with a broader range of services
Cultural expectations of the role of women		
Reluctance to engage with services		Enhancement of a service’s reputation within families or communities
Access to supportive family and social networks		
Level of trust between:		
-client and index case worker		
-client and client’s friend/family member		
-client’s friend/family member and case worker		
-professionals involved		
Whether client consent to refer to other services can be obtained		

The importance of wrap-around care was particularly evident when care-coordinators were seeking to engage with large kinship networks. Establishment of trust within families had a ripple effect, and could encourage other family and community members to seek out help from the HHAN team or associated services. This leveraging of existing relationships occurred both within family networks and within communities as a mechanism underlying effective wrap-around care.

“… the amount of people that walk in here and say such and such told me to come and see you….Because the word of mouth is being positive. So it's reputation and then there’s trust”. (Service provider)

The flip-side of leveraging of relationships with others was that an adverse interaction between a client or family and a care-coordinator could have far-reaching effects on trust across a wide social network. There were instances where it was difficult or impossible to engage others within the family and even direct conflict between care-coordinator and contacts. This was a particular issue in families affected by domestic violence or from cultures where women were devalued and the care-coordinator was female.

“I’m doing it to make a difference for those children and maybe to empower her…. To see that it isn’t ok for her kids but then you know he [her husband] will totally, totally hate us for that. We certainly don’t have his agreement for any [of her care] goals. And he won’t even agree to meet”. (HHAN CC)

Wrap-around care could also operate through leveraging collaborations between service providers, at the Intermediate Level. Relationships with one professional could act as a conduit to engaging clients with other services. Sometimes the trusted professional would act as a link between their client and other services, directly referring them on. At other times, they would consult other services, but continue as the sole person working face to face with the client. These leveraging approaches enhanced client outcomes by improving their access to a broader range of services without the need for clients to build trust with new workers.

Several professionals raised concerns about maintaining confidentiality when collaborating with other services, and obtaining consent from clients. This was an important contextual factor with the potential to mediate the impact of leveraging as a mechanism. NSW Child Protection Legislation (Section 16A, Children and Young Persons (Care and Protection) Act 1998), allows for information sharing between agencies without client consent, where there are concerns about the welfare of a child. In the current study, however, neither professionals nor clients gave examples of where they had experienced difficulties or complaints related to privacy.

##### Meeting the client on their own terms (Table 3)

Attending an appointment in an “institution” could be intimidating for somebody with low literacy or self-confidence, especially if they had had adverse experiences with authorities in the past. This was articulated by one educator working in a deprived social housing estate.

**Table 3:** CMO configurations “Meeting the client on their own terms”

Contextual factors	Mechanism	Outcomes
Presence of intergenerational trauma	Flexible service delivery by care coordinators	Client priorities are reflected in initial goal setting
Extent to which flexible service delivery is built into the programme		Clients’ needs are met
		Enhanced client engagement
Level of case worker autonomy in selecting approach		Staff stress and burnout prevented
Level of case worker willingness, patience and confidence to deviate from standard practice		
Existence of Occupational Health and Safety concerns		
Availability of other services that can assist with providing care and support CC’s role		
Level of trust between service providers

“Their priority is to help their child but they can’t actually get them to the Dental Hospital, even though it’s close, because they’re having a really hard time and they need support… some of them need hand-holding and confidence is a huge problem; we see parents when they first come here, they’re quite withdrawn and hollow and it’s like an institution…”

Meeting the client on their own terms through flexible service delivery was a crucial mechanism promoting engagement (see Table 3) that came up frequently during interviews with service providers and clients. Care-coordinators described adopting various non-standard interventions to promote engagement. Care-coordinators were often required to make themselves available at varied times and places, using the means of communication that was most acceptable to the client.

Genuinely listening to clients’ concerns and prioritising them in goal setting, particularly in the initial phase of involvement, was an important outcome of the flexible approach. By focusing on the client’s most pressing need, care-coordinators could build trust and create opportunities to subsequently work on other issues that they considered important. The success of this approach, however, hinged on care-coordinators’ willingness to be patient and flexible.

The down side of flexible service provision was that it could take its toll on care-coordinators, some of whom displayed evidence of burnout. Staff turnover could have a major impact on client outcomes, particularly continuity of care, trust and engagement.

“One of my patients who’s really difficult was doing great when he had a case worker…. Four months later he just DNAs [does not attend appointments] again, I can’t get hold of him and it’s because the case worker changed… I would have loved them to call me and let me know… then the patient ends up in hospital and then it can take six months to recover from those sorts of setbacks”. (GP)

At times, flexible service delivery was dependent on contextual factors at the Intermediate Level, such as the availability of other suitable services to assist clients.

“There can be services but a lot of them are at capacity… so we are often holding them”. (HHAN CC)

The intended role of HHAN care-coordinators was putting services and clients in touch with one another and arranging care, before gradually reducing intensity of involvement. This model of care proved difficult at times due to tensions between “care coordination” and “case management” roles. Care-coordinators were often required to be a lot more “hands on” than anticipated. Having built a relationship with the client, it could be difficult to step back (practically and emotionally). In several instances there were no services to take over care.

“I don’t see it’s possible to step away. So many of these clients have had no one to trust … I think it’s very important for these clients to know that there is someone out there who is stable, who isn’t judging them for whatever situation comes up next but simply helps them to navigate whatever challenge has come up next”. (HHAN CC)

##### Building trust (Table 4)

Many clients indicated that care-coordinators needed to be likeable, approachable and a “safe person”. Without these qualities, a relationship of trust would not be achieved. Care-coordinators wore casual clothing, used informal language and avoided jargon to erode the notion of a power base and maintain approachability.

A key challenge to building trust was that several clients had previous or ongoing involvement with child protection services. Although not directly involved, care-coordinators remained bound by mandatory reporting obligations. In Australia, all health staff are required by law to report suspected cases of child abuse or neglect to the relevant government authority. In NSW, mandatory reporting is regulated by the Children and Young Persons (Care and Protection) Act 1998 (the Care Act) [54] and mandatory reporters are guided by the NSW Mandatory Reporter Guide. Care-coordinators had to juggle their interactions with child welfare workers and the client and, at times, this resulted in conflict. One client disengaged as a result of child protection concerns reported by a care-coordinator.

**Table 4:** CMO configuration “Building Trust”

Contextual factors	Mechanism	Outcomes
Whether client had had a child removed from their care	CC likeable and approachable: “a safe person”	Level of client engagement
Level of distrust of authority		Level of CC burnout
Importance of making changes to retain custody to the client		
Presence of child protection concerns		
Relationship between CC and Family and Community Services		

##### Engaging by demonstrating effectiveness of the partnership (Table 5)

Clients frequently reported that trust in their care-coordinator was primarily based on the practical benefits that stemmed from engaging with the worker, such as being successfully rehoused, or accessing financial support. A number of contextual factors impacted, however, on whether or not care-coordinators could demonstrate their effectiveness. For example, clients frequently spoke of the importance of confidence in their care-coordinator’s skills and experience. All four HHAN care-coordinators were highly experienced nurses and social workers. Data suggested that clients may have been less likely to give a care-coordinator a chance to prove their worth if they were younger or less experienced.

**Table 5:** CMO configuration “Engaging by demonstrating effectiveness of the partnership”

Contextual factors	Mechanism	Outcomes
Clients’ past experiences with services and willingness to give CC a chance	CCs demonstrate effectiveness of partnership	Client engagement in HHAN
Complexity of client problems		Enhanced outcomes for client and family
Level of CC knowledge, experience and persistence in effecting positive change for client		
		Prevention of CC burnout
CC’s reliance on other services to provide care, and their capacity to meet the client’s needs		

In many cases the client’s problems were complex and unpredictable. In this context, care-coordinators needed to have realistic expectations of outcomes, and be adept at navigating which problems could be solved.

“Literally every week it’s a major crisis…who’s broken into the house, who’s lost what, who’s hit who this week… it’s just like a horror movie for some of these people”. (Stakeholder)

As discussed previously, Intermediate Level factors such as the availability of other services, shaped the mechanisms and subsequent outcomes. In addition, when care-coordinators were dependent on other services to assist the client, and the intervention was unsuccessful, the relationship with the client and care-coordinator could be jeopardised.

##### Making clients feel “valued and empowered” (Table 6)

Promoting client independence was a key outcome of client empowerment.

**Table 6:** CMO configuration “Making clients feel valued and empowered”

Contextual factors	Mechanisms	Outcomes
Level of client confidence given history and current social situation	CC demonstrates valuing of client by advocacy and “going beyond the call of duty”	Promoting client independence
Level of client motivation		Client empowerment
Client’s view of authority figures		Enhancement of client outcomes
Level of responsibility CC gives to client		
		Prevention of CC burnout

“The aim is hopefully that we’re not babysitting families… we’re trying to promote independence so they feel comfortable, connected with community and health”. (HHAN CC)

All care-coordinators described taking active measures to avoid creating dependency in their clients such as intentionally making clients call to make appointments for themselves. Wherever possible, they took on a ‘guiding’ rather than a ‘doing’ role.

“She’s given me some guidance, I’m familiar with where I should go now and what needs to happen … I’m comfortable doing so”. (Client)

Client dependency could result in care-coordinators feeling isolated. In the case of more challenging clients, dependency could also lead to staff burn out.

“For most of my clients it’s taken so long to find someone who fits their needs because there’s such a broad spectrum that somebody requires, the clients don’t let go. I’m still in there, they’re still ringing me, they’re still having this relationship with me where they will tell me about the ongoing problems. I think that’s a good thing because they’re still opening boxes for me and I’m finding other things that are still layer upon layer… but it’s also really challenging because you don’t kind of get a break with these clients. You don’t have the easy clients which offset the more challenging clients; they’re all challenging”. (HHAN CC)

Although no adverse effects of burnout were reported by clients, it was anticipated that care-coordinator burnout and stress might threaten the relationship with the client and the longevity of the programme.

“I really struggled within this job for quite a period of time because it was nothing like I thought it would be … as I actually developed my own sense of what my role within the team was”. (HHAN CC)

#### Strata: intermediate level – integrating care

##### Knowledge transfer (Table 7)

As discussed, the HHAN intervention was designed to build capacity for interagency collaboration. Interview data highlighted that key mechanisms for enhancing staff collaboration related to knowledge transfer activities.

**Table 7:** CMO configuration “Knowledge Transfer Activities”

Contextual factors	Mechanisms	Outcomes
Co-location of service providers	Knowledge transfer between staff working together	Shared goals, language and professional learning activities among collaborating interagency staff.
Physical proximity of collaborating staff		
Willingness of CC to work with others		Faster and more appropriate resolution of client problems
Existing relationship between CC and other staff in inter-disciplinary and inter-agency teams		Enhanced CC decision-making and work satisfaction
		Reduced conflict between agencies
Links between CC and other services		Capacity to enhance outcomes
Willingness of staff to share knowledge		Relationship building among workers

HHAN adopted a model of staff collaboration and inclusivity in which professional learning pathways and training opportunities were created and shared across different disciplines and organisations. Knowledge transfer activities and shared learning were both formal and informal, including multidisciplinary meetings, clinics and training sessions. Professional partners focused on shared goals and language in order to further their projects and avoid conflict.

“We are certainly taking this global approach. This brings global problems and knowing how to support both clients and the staff through this process has been challenging”. (HHAN CC)

“We’ve all grown as a result of it and I think we’ve become less protective or defensive of our roles as a nurse or a social worker, you know, I feel more comfortable with being part social worker now despite the fact I’ve had no formal training in it”. (HHAN CC)

Service providers almost universally described co-location with other services as extremely helpful for integrating care. Co-location promoted regular informal knowledge transfer and offered opportunities for enhanced collaboration.

“Instead of doing it by myself and having to make calls, I have access to learned professionals in different areas such as legal, drug health, youth services, health and family services, paediatrics, all surrounding and supporting me.... you could have a genuine real time collaboration within minutes of engaging with a client and you could also wrap- supports around a client in real time, that's the biggest difference. I have found that better outcomes for the client and the actual timing is just so much quicker …. Now that I have access to my own advice, my decisions are far better”. (Stakeholder)

##### Implementing structural change (Table 8)

HHAN challenged traditional welfare provision to disadvantaged families. Siloed funding and management structures, and resistance to collaboration among service providers, were key contextual factors influencing the mechanisms related to integrated care.

**Table 8:** CMO configuration “Implementing structural change”

Contextual factors	Mechanisms	Outcomes
Level of insight or awareness of difficulties faced by other service providers	The desire to bring about positive change	“Systems thinking” among professionals
Service provider resistance to collaboration		Creative problem solving
Acknowledgement by professionals of systemic barriers to care		Actively seeking to connect with unknown service providers
Extent of buy-in from managers regarding integrated care		Strengthening and simplifying of referral processes
Siloing of funding sources		Enhanced staff knowledge of other services’ roles
		Shared training opportunities for staff from different agencies

Simplifying referral processes resulted in increased outcomes and satisfaction for both clients and stakeholders. The HHAN programme director and manager required support from partners and stakeholders for this to occur. In some instances, there was a lack of understanding of the roles of other professionals and an inability to see benefits of collaboration.

“There has been a lot of shakeup…I think even the importance of highlighting those silos has been really important and those conversations but...I know how difficult that change can be, culture change and how people are quite fearful of those changes… that pushback can be quite strong”. (Stakeholder)

In order to promote care integration and structural change, traditional power bases had to be challenged, in a respectful and constructive manner, ensuring all professional opinions were respected.

“I really do believe…it’s bringing all the agencies together to brainstorm how we’re going to and listening to people’s expertise around the table”. (Stakeholder)

##### Fostering mutual respect and trust (Table 9)

Inter-agency guidelines and agreements were sometimes used to build consensus between staff and agencies. HHAN was also able to empower some services by validating them in the eyes of others.

**Table 9:** CMO configuration “Fostering Mutual Respect and Trust”

Contextual factors	Mechanism	Outcomes
Attitudes towards collaboration among staff	Advocacy for, and validation of, other professionals or agencies	Challenging of traditional power bases
		Guidelines and formal agreements to build consensus
Service provider knowledge of other services’ roles		
		Enhancement of inter-agency relationships
Differing world views and personality clashes		
		Potential for shifting staff attitudes towards collaboration through positive experiences
Physical proximity of staff working together		
		Increased likelihood of working together in the future
Extent to which HHAN were likeable, available and persistent		
Management support for collaboration

“I’ve been knocking on one organisation’s door for a very long time… a great thing about HHAN is that if there’s been an introduction there so they’ve helped me link to that organisation…. Because health [HHAN] is there standing beside us saying we want you there to help”. (Stakeholder)

HHAN staff made themselves approachable and available, and were persistent in attempting to develop and strengthen relationships, even in the face of inevitable conflicts. The process of shared learning and team troubleshooting in relation to difficult clients, served as opportunities to foster trust and respect between service providers.

“The HHAN director is always available … I think that willingness, that relationship and the respect for the services I think is what pushes this project along in my view”. (Stakeholder)

The HHAN team and stakeholders described many contextual factors influencing the extent to which respect and trust could be fostered among service providers. Different organisations and individuals often had opposing worldviews. In some instances, personality clashes between individuals could have a particularly destructive flow-on effect.

“I know she has felt that I shouldn’t be at the meetings… I heard that an email was sent saying it had been decided that I should not attend those meetings. [it] really disrespects me as a person and as a professional”. (HHAN CC)

##### Cultivating a culture of faith in positive change (Table 10)

In order to create sustainable positive change in relation to integrating care, HHAN staff had to change cultures both within and between organisations. Service providers had to role model collaboration and become advocates for the HHAN programme in the hope that they would win over other hearts and minds. There was evidence of transformational leadership at all levels.

**Table 10:** CMO configuration “Cultivating a culture of faith in positive change”

Contextual factors	Mechanism	Outcomes
Staff burnout and jadedness	Cultivating faith in positive change related to integrated care	Enhanced buy-in by staff and agencies
Visibility of benefits of collaboration		Role modelling collaboration
		“Selling” the benefits of HHAN through advocacy
Realistic expectations of time frames of change		
		Experiential learning
		Creative problem solving Enhanced resources for implementation
Presence of inspiring transformational leadership		
		Quality of staff involved
		Level of staff turnover

Service providers sometimes reported that “selling” HHAN to other services could be challenging unless they could demonstrate the positive benefits of the initiative.

“It seems that it wasn’t a very easy exercise to get people to agree to what you are doing… there was an awful lot of explanation of who we are”. (HHAN CC)

In the case of complex interventions like HHAN, it could be difficult to clearly demonstrate benefits as it is hypothesised that change occurs over a long period of time. As one care-coordinator noted:

“We’re learning as we go. I do think we’re making a difference in people’s lives. Sometimes the case studies show you that much more”.

The risk of workers becoming burnt out and jaded posed a threat to the project. The provision of ongoing practical and moral support to all staff was therefore crucial.

Moreover, the project was large and ambitious. Different parties seemed to have different expectations of what HHAN would be able to achieve and over what time frame. In some instances this mismatch of expectations resulted in disappointment and conflict.

“I just had a hope that this time it would move a bit quicker. I don’t think you can actually judge it yet. I think to try and judge it now is too soon. Honest to goodness it needs more funding and it needs a longer period of time… I don’t know what people were expecting from HHAN either. If they think you’re going to get dramatic change in an instant then you know it never it works like that”. (Stakeholder from NGO)

## Discussion

Critical realist research and evaluation methodology is increasingly being used in the study of health and social care systems including endeavours aimed at improving the integration of care delivery. The realist approach to implementation science is to look ‘beneath the hood’ and to ask the questions how and why. The critical realist approach places great importance on the layered nature of the study context (C) and seeks to understand how underlying mechanisms (M) interact to produce the observed or experienced outcome (O) phenomenon. The study of these CMO theoretical configurations can greatly assist the explanation of programme outcomes within context, and thus assist programme improvement. It is here that this study will contribute to the future shape and development of HHAN. The study was undertaken at the feasibility/piloting phase of the HHAN development and evaluation process and will inform the design of the next phase in terms of both intervention elements and the conceptualisation of programme theory.

Integrated care can be polymorphous in nature, as it follows a variety of frameworks and models, making it difficult to implement, compare outcomes and identify enablers. Previous studies suggest integrating care has particular benefits for people with complex health and social needs but have argued that more research is needed to identify specifically how and why [11]. There have been few previous realist studies of the impact of integrated care interventions on vulnerable child and family populations. Implementation evaluations of integrated care, and systematic reviews of those interventions, do not seek to explain the intervention outcomes or underlying context and mechanisms [33, 55–59]. The recent systematic review by Wolfe and colleagues [60], for example, reported on quality of life and emergency department visit outcomes, but did not seek to explain those findings through the use of realist methods. By contrast, Tyler and colleagues [61], in their realist synthesis, identified four patterns of care that may be effective, namely: “1) horizontal partnerships based on willingness to share status and power; 2) bridged trust initiated through previously established third party relationships; 3) knowledge support increasing providers’ confidence and skills for engaging community; and 4) increasing vulnerable families’ self-reliance through empowerment strategies”. The findings reported here are consistent with those of Tyler and colleagues, and contribute to an emerging literature regarding the key components of effective integrated care initiatives for vulnerable populations. In this study we have particularly focused on two layers of the programme theory, namely face-to-face activity – engagement of vulnerable families, and the intermediate level – integrating care.

Achieving engagement of vulnerable families, who often distrusted service providers and resisted engagement, was fundamental to enhancing family outcomes and increasing their access to a broader range of support services. Active involvement of care-coordinators is a known component of effective collaborative care [62], however, there is often ambiguity and confusion about the role of a care-coordinator [63], with limited consensus on the essential components of care coordination [64, 65]. The study highlighted the crucial importance of care-coordinators having sufficient time, autonomy, skills and experience, flexibility and patience to build trust and credibility as a valuable resource for this group of clients which is not always explicitly outlined in models of care coordination. This study also adds depth to the existing literature which mostly describes care coordination for individuals with chronic disease, by describing the ecological contextual factors which are themselves adding further complexity to the provision of care coordination services to vulnerable people. Client motivation and readiness to make changes, access to supportive family and social networks, as well as the presence of trauma, substance use issues, child protection concerns, domestic violence and cultural norms where women are devalued, influenced mechanisms and outcomes. These findings suggest the long-term nature of effective service provision may prove resource intensive and reinforces the need for further research into the cost benefits of integrated care initiatives [11, 12].

As described by others [62, 64], integrated care was found to be optimised by trust among service providers, willingness to collaborate, knowledge of other services’ roles, shared interagency learning opportunities, co-location of collaborating staff, management support of collaboration, and the presence of transformational leadership. Care-coordinators having realistic expectations for change and levels of stress and burnout were also directly aligned with the potential for mechanisms to lead to positive outcomes. Service providers could be exposed to high workload, vicarious trauma, dis-spiriting pushback or conflict with other agencies and the need to work outside their comfort zones. Burnout and staff turnover could jeopardise the project as a whole, reinforcing the need to examine the resource demands of this approach and how to institute sustainable work and staff support practices. Ensuring that care-coordinators are contributing to system-change projects, as well as work with individual patients, may foster sustainability [66], and complement other strategies to ensure staff wellbeing such as training, supportive supervisors and managers, and a supportive team environment [67].

### Strengths and weaknesses

The study triangulates the perspectives of clients and service providers and adopts a realist approach to explore contexts, mechanisms and outcomes associated with the effectiveness of HHAN. The research also highlights the need to change organisational cultures, challenge traditional power bases and integrate funding sources to reduce siloing and improve access to care for vulnerable families. However, the study also had limitations. Obtaining consent for interviews for clients was challenging and affected sampling and recruitment. It is likely that those participants who were interviewed were the most confident and articulate clients, and those with a positive attitude towards HHAN. Access to the most vulnerable clients might have given a different and extremely valuable perspective. In addition, analytic processes adopted for development of CMO configurations were inherently vulnerable to subjectivity and may have resulted in misattributions regarding causality.

## Conclusions

This paper adds to an emerging evidence base for the use of critical realist approaches in translational social epidemiology. The research confirmed and further refined HHAN’s programme theory in terms of how the service engages with clients and seeks to integrate care provision serves to enhance outcomes for families with complex needs. Breaking the intergenerational cycle of disadvantage requires innovative and more nuanced approaches to designing and evaluating complex interventions, such as those targeting vulnerable populations with a plethora of health and social needs. Findings from this pilot of the HHAN Integrated Care Initiative reinforce the multi-dimensional nature of factors implicated in an intervention’s impact and the potential constraints they pose for translation into practice in different settings. This research highlights that programme outcomes remain contingent on enabling contexts that set in motion the mechanisms that will lead to success.

## Supplementary information

Additional file 1: **Appendix 1.**Theory of Change Model for Healthy Homes and Neighbourhoods Integrated Care Program, Sydney, Australia. (DOCX 479 kb)

Additional file 2: **Appendix 2.** Initial Programme Theory for Healthy Homes and Neighbourhoods Integrated Care Program, Sydney, Australia [14]. (DOCX 22.1 kb)

Additional file 3: **Appendix 3.** Questions for Guided Interviews. (DOCX 18.8 kb)

Additional file 4: **Appendix 4.** Healthy Homes and Neighbourhoods Summary Logic Model. (DOCX 41.9 kb)

## Data Availability

The datasets generated and/or analysed during the current study are not publicly available due the risk this poses to the confidentiality of participants. However, de-identified data may be available from the corresponding author on reasonable request.
