# Supplementary material for: A critical realist evaluation of an integrated care project for vulnerable families in Sydney, Australia
Source: BMC Health Serv Res. 2020 Oct 31;20:995. doi: 10.1186/s12913-020-05818-x (PMC7603742; doi:10.1186/s12913-020-05818-x)

## Appendix 1: Theory of Change Model for Healthy Homes and Neighbourhoods Integrated Care Program, Sydney, Australia


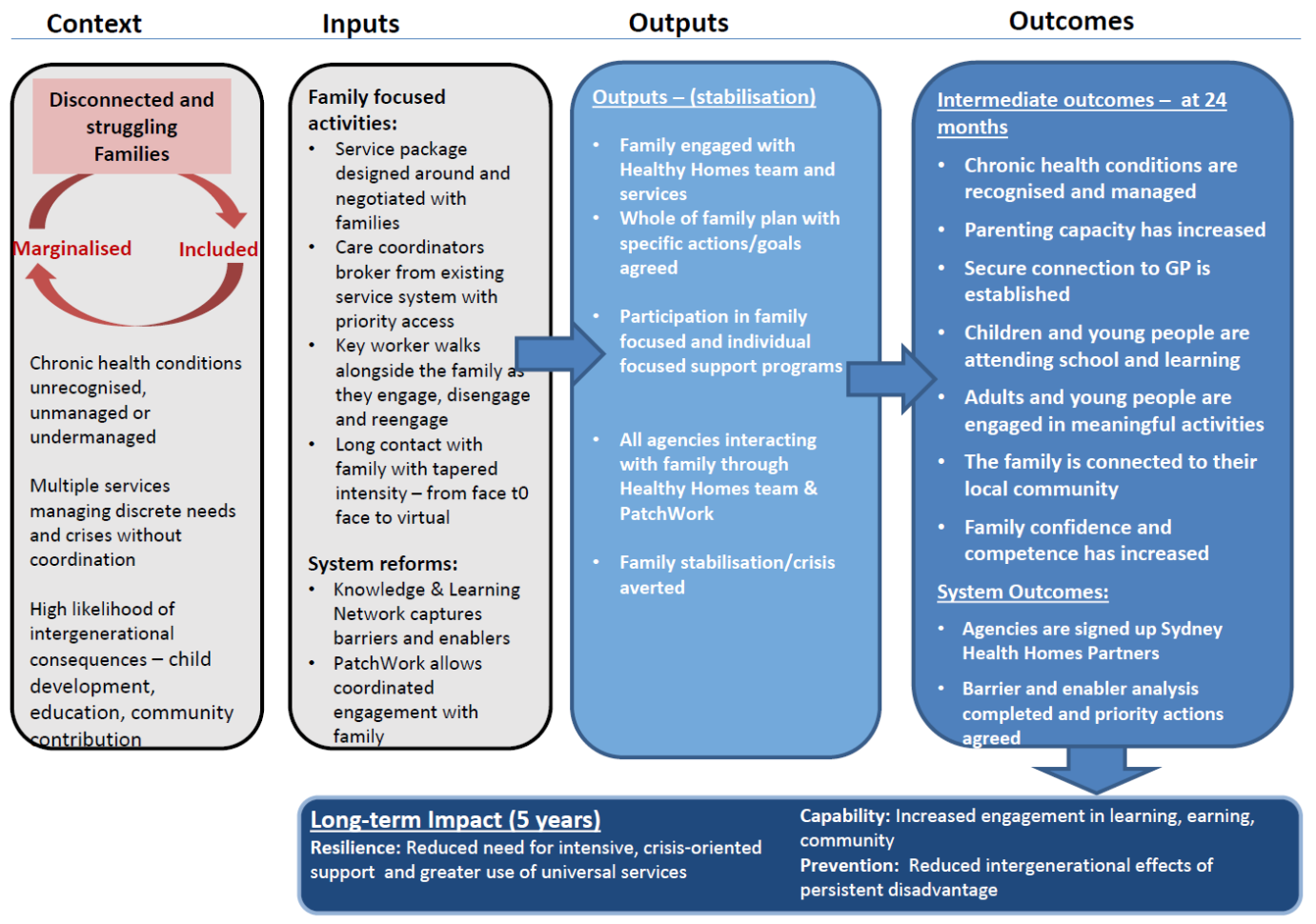

Supplement: Supplementary file 1 — Appendix 1.Theory of Change Model for Healthy Homes and Neighbourhoods Integrated Care Program, Sydney, Australia. (DOCX 479 kb) [file 12913_2020_5818_MOESM1_ESM.docx]
