# Supplementary material for: A critical realist evaluation of an integrated care project for vulnerable families in Sydney, Australia
Source: BMC Health Serv Res. 2020 Oct 31;20:995. doi: 10.1186/s12913-020-05818-x (PMC7603742; doi:10.1186/s12913-020-05818-x)
Supplement: Supplementary file 2 — Appendix 2. Initial Programme Theory for Healthy Homes and Neighbourhoods Integrated Care Program, Sydney, Australia [14]. (DOCX 22.1 kb) [file 12913_2020_5818_MOESM2_ESM.docx]

## Appendix 2: Initial Programme Theory for Healthy Homes and Neighbourhoods Integrated Care Program, Sydney, Australia [14]

|  |  |  |  |  |
| --- | --- | --- | --- | --- |
| Theorised Contextual Conditions (Fig. 2)  [C] | Present contextual mechanisms activated  [CM] | Proposed Intervention Design Elements (Table 1)  [I] | Postulated Intervention Programme Mechanisms  (Table 1)  [MP] | Postulated psychological, motivational and behavioural Outcomes  [O] |
| Self – Self-identity and individual’s experience | | | | |
| Lack of partner and family support,  Distrust of services,  Limited treatment access | Stress mechanism activated causing anxiety and depression | Friendship and family support, Professional support, Medication, Treatment | - Activate mediating mechanisms of family, peer and professional support to strengthen and build trusting relationships with peers, family and clinicians through SHHV and FCISD Design Components. | Decreased depression and anxiety |
| Lifetime trauma, Loss, Being alone, Isolation | Stress mechanism activated arising from mismatched expectations, and loneliness | Family and peer support,  Home visiting, Telephone support |  | Increased perceived support |
| Situated Activity – Face to Face activity | | | | |
| Services unavailable or poor access,  Services not trusted,  Services not skilled | Absence of trusted professional support mechanism | “wrap-around” services,  Family Conferences, Workforce training | - Activate services mechanisms that are client, peer and neighbourhood focused, and trauma and evidence informed through FCISD and IS Design Components. | Improved perceived access to skilled and trusted services |
| Community distrust, Low social capital and cohesion, crime, unemployment | Absence of trusted neighbourhood and community support mechanism | “wrap-around” services,  Family Conferences, Public health,  Social work services |  | Improved perceived support from neighbours and community |
| Intermediate Level social and service organisation | | | | |
| Unhelpful intake and referral practices, Lack of service, knowledge and trust | Absence of specialist service support mechanism for front-line professionals | Strengthened pathways and design  Collocation of services | - Activate mechanisms related to trust and confidence with service network, increased local social capital, community trust and community safety - Activate mechanisms relating to improved coordination and access to services and information through FCISD and IS Design Components. | Improved perceived access to services that are “wrapped” around front-line workers |
| Weak social networks, community trust, community safety, available social services, access to information | Social level stress mechanisms relating to class, position, racism, segregation, crime and neighbourhood decay are activated tending to increase psychological stress | Population and community level interventions in neighbourhoods and communities |  | Decrease in psychological stress of individuals and families |
| Macro Level social and service organisation | | | | |
| Migration, Mega-malls pull service activity away from neighbourhoods,  Urban development | Activation of social level stress mechanisms tend to hinder the activation of social level buffer mechanisms | Population and community level interventions in neighbourhoods and communities | - Activate mechanisms related to increased social level activities in deprived neighbourhoods. - Activate mechanisms related to increased migrant related social activities among ethnic populations through FCISD and IS Design Components. | Increase in perceived social level buffers |
| Immigration policy, Racism, Media policy, Global market, Settlement patterns, Ethnic bonding networks, Access to services | Migrant related social level mechanisms including acculturation, cultural practices and integration tend to decrease social level stress | Ethnic and cultural specific community and population level interventions |  | Increase in perceived migrant social level buffers |

Note: SHHV-Sustained Health Home Visiting; FCISD – Family and Community Integrated Service Development; IS-Infrastructure Support
