# Supplementary material for: A critical realist evaluation of an integrated care project for vulnerable families in Sydney, Australia
Source: BMC Health Serv Res. 2020 Oct 31;20:995. doi: 10.1186/s12913-020-05818-x (PMC7603742; doi:10.1186/s12913-020-05818-x)
Supplement: Supplementary file 3 — Appendix 3. Questions for Guided Interviews. (DOCX 18.8 kb) [file 12913_2020_5818_MOESM3_ESM.docx]

# Appendix 3: Questions for Guided Interviews

## Questions for clients

#### Contexts:

- Would you like to tell me a little bit more about your situation and why you sought help from HHAN? Has that situation changed during the time you’ve been involved?
- Have you found it easy to get the help/care you need in the past? Can you give me examples of where it has been easy/difficult?
- Can you give me examples of where having HHAN involved helped?
- Can you give me examples of where having HHAN involved did not help? Can you think of any ways where having the HHAN team involved did or could make things worse?
- What kind of people and in what kind of situations do you think the HHAN program might help?
- Are you happy for me to look at your notes/speak to your CC/speak to others in relation to this?

#### Mechanisms:

- How did you hear about HHAN?
- What made you agree to participate in/accept help from HHAN? (why)?
- Do you think that you would still be involved with HHAN if you had to travel for an appointment to see the team? (is it easy or difficult for you to travel to appointments? – what makes it difficult?)
- What things are important about the HHAN program? What do you like/dislike?
- Have you learned anything from HHAN?
- Has being involved with HHAN made you think differently about yourself and your family?
- I’m really curious about what it actually is that works about HHAN? What is it that gave you the confidence or the motivation to attend appointments/get out of the house etc.
- Has your relationship with HHAN affected your relationship with your children/how you feel as a person/how you feel about your role as a parent?

#### Outcomes:

- Since you had support from HHAN have you attended appointments with other carers/organisations that you wouldn’t normally have done? (why do you think this is?)
- Since you had support from HHAN has there been a change in how you feel in or about yourself? (Do you think this is because of HHAN or was anything else happening in your life that caused this?)
- Has being involved with HHAN changed your relationship with your children at all (if so, how?)
- Have you noticed any changes for you/your child/your relationships/your parenting /your relationships with your doctor/centrelink/other services since you became involved with HHAN?

## Questions for Stakeholders

#### Contexts:

- Please tell me a bit about yourself, your organisation and the kinds of clients you work with?
- What kinds of issues do your clients face?
- Do you think it is easy for vulnerable families to get the support they need? Why?
- What distinguishes clients who will benefit from HHAN from those who will not benefit from HHAN? Can you give me examples?
- Have there been external factors that have helped your relationship with HHAN or made it more difficult?
- Are there features of the HHAN program that work in some situations but not others? Can you tell me some examples?
- Can you give me examples of where having HHAN involved helped?
- Can you give me examples of where having HHAN involved did not help? Can you think of any ways in which having the HHAN team involved can make things worse?
- What kind of people and in what kind of situations do you think the HHAN program might help?

#### Mechanisms:

- How did you hear about HHAN?
- What made you agree to partner with HHAN?
- Do you think that you would still be involved with HHAN if you were not based in the same office (for Redlink)?
- What things are important about the HHAN program? What do you like/dislike?
- Have you learned anything from HHAN?
- Has being involved with HHAN affected your relationship with other service providers?
- I’m really curious about what it actually is that works about HHAN, or doesn’t work?
- Has your relationship with HHAN affected your relationship with your clients at all?

#### Outcomes:

- Have your clients been more engaged in care since they were involved with HHAN?
- Has your clients’ health improved since being involved with HHAN?
- Do you have a better relationship with other care providers since your involvement with HHAN?
- What changes have occurred for you and your clients since your involvement with HHAN?
- Please can you give examples to explain your answers to the questions above?
