# Supplementary material for: A critical realist evaluation of an integrated care project for vulnerable families in Sydney, Australia
Source: BMC Health Serv Res. 2020 Oct 31;20:995. doi: 10.1186/s12913-020-05818-x (PMC7603742; doi:10.1186/s12913-020-05818-x)
Supplement: Supplementary file 4 — Appendix 4. Healthy Homes and Neighbourhoods Summary Logic Model. (DOCX 41.9 kb) [file 12913_2020_5818_MOESM4_ESM.docx]

**Context**

**Interventions**

**Component 1**

**Identification**

**Outcomes
(∆Causal Mechanisms)**

**Mechanisms
(Program Mechanisms)**

*Consumer Level*

- Disconnected and struggling families
- Chronic parent health conditions unrecognised & unmanaged
- Marginalised families
- Lack of trust in services
- Intergenerational consequences for child development, education
- Clusters of locational disadvantaged families

*Provider Level - Health*

- Antenatal screening and pathways poorly implemented
- Teen and Aboriginal SHV in place with limited coverage
- No SHV for other groups
- Limited HV for most need
- CE support for Tiered model of SHV
- Tier 2 services limited in link to SHV and no central intake system
- Very limited universal well child & family services
- Limited services for families with most need
- Multiple services managing discrete needs and crises without coordination

*Services Level*

- Strong interagency collaboration and planning
- Interagency parent communication initiative
- Strong school and local government engagement

**Design Component**

Clinical Care

- Strengthen existing identification and referral pathways through review, training and digital tools
- Implement population level high risk family care coordination and cohort tracking
- Integrated service models including wrap-around and family group conference model
- Parening Programmes

*Consumer Level*

- Family – provider trust
- Family – peer trust
- Provider willing to share power with consumer
- Sharing of information
- Building of self-help skills

*Provider Level*

- Service providers share information and power
- Shared policies, standards, protocols
- Shared assessment tools

*Service System Level*

- Shared vision at the agency level
- Shared outcome framework
- Information sharing protocols
- Resources shared
- Training opportunities shared

*Consumer Level*

- Increased mastery
- Increased sense of control
- Increased support
- Realistic expectations implemented
- Increased knowledge and confidence in ability to provide care to child and self

*Provider Level*

- Improved provider engagement with families
- Improved provider collaboration
- Increase shared care

*Service System Level*

- Improved agency collaboration
- Improved trust between agencies

Provider Capacity

- Shared design and implementation of evidence-informed interventions including parenting
- Capacity building of service network including eLearning, web-based and mobile technology
- Support for general practice
- Immunisation and Healthy Housing
- Healthy lifestyle initiatives

**Component 2**

**Care-Coordination**

**Component 3**

**Evidence-Informed**

**Component 4**

**General Practice**

**Component 5**

**Family Health**

**Component 6**

**Place-based**

**Component 7**

**System Change**

**Component 8 & 9**

**Outcomes & Evaluation**

System Change

- “Hub” and “place-based” community building and service coordination
- Project management and leadership
- Sector capacity building projects
- System change projects
- Child and Family public health (research, program, evaluation)
